# Microbial and Antibiotic Susceptibility Profile among Clinical Samples of Patients with Acute Leukemia

**Published:** 2016-04-01

**Authors:** Alireza Abdollahi, Faezeh Hakimi, Mahsa Doomanlou, Azadeh Azadegan

**Affiliations:** 1 Associate Professor of Pathology, Department of Pathology, School of Medicine, Imam Hospital Complex, Tehran University of Medical Sciences, Tehran, Iran; 2 Associate Professor of Pathology, Thrombosis Hemostasis Research Center, Tehran University of Medical Sciences, Tehran, Iran; 3 Department of Pathology, Imam Hospital Complex, Tehran University of Medical Sciences, Tehran, Iran; 4 MSc in Microbiology, Central Laboratory, Imam Hospital Complex, Tehran University of Medical Sciences, Tehran, Iran; 5 MSc in Laboratory Medicine, Central Laboratory, Imam Hospital Complex, Tehran University of Medical Sciences, Tehran, Iran

**Keywords:** Acute leukemia, Antimicrobial susceptibility, Clinical fluids, Microbial profile

## Abstract

**Introduction:** Preventing and starting early treatment of infections in patients whose immunity system is weak due to malignancies like leukemia can reduce mortality. This study aimed to determine microbial and antibiotic resistance patterns in clinical samples of patients with acute leukemia to start early treatment before the results of clinical tests are known.

**Subjects and Methods:** In this cross-sectional study, the clinical samples of all patients hospitalized with the diagnosis of acute leukemia were cultured and their antibiogram was evaluated. Then, the data were analyzed by SPSS 18 based on the objectives of the study.

**Results:** Of a total of 2,366 samples, 18.95% were reported to be positive blood samples, 22.96% were reported to be urine samples and 36% wound samples. E. coli was the most common bacteria isolated from the blood and urine cultures (34% in blood, 32% in urine culture) while Staphylococcus Aureus was the most common in the wound culture (35%). The highest level of sensitivity in the organisms with positive blood culture was to Ciprofloxacin, while in positive urine and wound culture was to Imipenem. The highest resistance in blood, urine and wound culture was to Cotrimoxazole.

**Conclusion:** According to results obtained from this study, it is necessary to conduct appropriate studies on this issue in specific conditions in our country. The findings of this study can be used in clinics for more accurate diagnosis, more effective treatment before the results of clinical tests are known and also for prevention of infection in cancer patients.

## Introduction

 Cancers are one of the top global hygiene and health issues after cardiovascular diseases, traumatic accidents and major crises in the world.^1^ Cancer caused about 13% of all human deaths worldwide.^1^ More than half of all cancer cases and about 60% of deaths occur in developing countries.^1^^-^^3^ Leukemia and lymphoma are neoplastic diseases that affect hematopoietic and immune systems. These diseases represent with different forms of clinical and pathological manifestations.^2^^-^^4^ The prevalence and incidence of leukemia and lymphoma are noticeably significant both in Iran and around the world, especially in the northern regions of Iran. Common types of leukemia are acute lymphocytic leukemia (ALL), which is especially common in children and acute myeloid leukemia (AML). These diseases occur in both genders and at any age; however, the incidence of this disease is more common in male individuals.

Infection is one of the most serious complications and the leading cause of morbidity and mortality in patients with acute leukemia receiving chemotherapy.^3^^-^^6^ Several factors increase the risk of infections in cancer patients whose immune systems were weakened. These factors include neutropenia during the anticancer treatment, changes in intestinal microbial flora due to multiple antibiotic medications and destruction of the skin defense mechanism and damaged epithelial surfaces due to cytotoxic agents.^6^^-^^7^ Some studies were conducted on main microbial causes and susceptibility of these microorganisms to certain antibiotics in cancer patients, especially in the patients with acute leukemia, in most parts of the world but few investigations have been held on the microbial pattern and their antibiotic susceptibility in leukemic patients in IRAN and our region.

E-coli, Stenotrophomonas Maltophilia, Staphilococcus Aureus are identified as the most common causes of infections in these patients.^4^^-^^9^ However, it is clear that the microbial and the antimicrobial susceptibility and resistance patterns would be different according to health and hygiene issues in every community.

Antibiotic treatment can be administered once the symptoms of infection were manifested on the conditions that the common bacterial agents causing infection in these patients as well as their antibiotic sensitivity and resistance patterns were known. Administration of antibiotic treatment at the beginning of manifestation of symptoms of the disease can even prevent the spread of the disease and may ultimately reduce rates of deaths, disabilities and complications due to cancer. Hence, this study aimed to determine microbial and antibiotic resistance patterns in clinical samples of a large group of patients with acute leukemia.

## SUBJECTS AND METHODS

 This cross-sectional study was conducted in Imam Hospital complex, Tehran University of Medical Sciences, between Nov. 2013 and Nov. 2014.The study included patients with acute leukemia (ALL, AML) admitted to the hematology and oncology ward. Patients with nosocomial infection (infections manifested 48 hours after hospitalization) were excluded. All patients were provided with the necessary information relevant to the procedure of the study before they accepted to participate in the study. They were fully informed of both the process and various steps to the study. Patients were assured that all their personal information would remain confidential. Moreover, this study did not impose additional costs on the patients for diagnosis and treatment. The Medical Ethics Committee of Tehran University of Medical Sciences confirmed the procedure of this study.

Demographic characteristics such as age, gender and underlying disease type were recorded. The blood, urine and wound/lesion samples were collected from the patients. Then, these samples were cultured in accordance with the guidelines of CLSI and standard methods in the central laboratory of the hospital complex.

 **Samples**

**Blood:** The blood samples taken from the patients were injected in the Thio medium. BHI or TSB (small vials) was used for the children because fewer blood samples were required to be injected in the thio medium.Then, the blood samples were sent to the microbiology department. In this department, the samples were transferred to incubator. After 24 hours, the samples were cultured on EMB media at first. Then, a spread of the cultured sample was collected from each vial for gram stain and microbial assessment. After 48 hours, the same task was repeated to detect whether any bacteria were present in the culture. If any microorganism was detected in the sample, it was evaluated further in order to assess the type of bacteria.

**Urine:** Urine cultures were performed by the calibrated loop. This loop was sterilized with appropriate heat temperature. The urine sample was well mixed in the container. Then, the loop was entered the container perpendicular to mix urine. Afterwards, the loop was removed; the sample was taken and cultured on the blood agar medium.

The same procedure was repeated to culture the sample on Mac Conkey medium. Then, the plates were transferred to the incubator, incubated for 24 hours at 35˚ and examined whether any microorganism had grown on the medium culture.

**Wound:** The wound’s exudates and festers were inoculated on the plates. Then, they were cultured. The direct slide was prepared using Gram stain. After 24 hours incubation or 24 hours of incubation, the media was removed from incubator. Then, they were examined whether any microorganism had grown on the medium culture.

**Antimicrobial susceptibility testing**

The Kirby-Bauer technique (Disc Diffusion Method) was used. Several antimicrobial disks were placed on the surface of Muller-Hinton agar plates followed by incubation at 35 °C. Reading the plates was carried out after 24 hours using transmitted light by looking carefully for any growth within the zone of inhibition. Standard quality control ATCC strains with known minimum inhibitory concentration including Staphylococcus Aureus ATCC 25923, Escherichia Coli ATCC 25922 and Pseudomonas Aeruginosa ATCC 27853 were involved in each run. Standard biochemical tests performed according to clinical and laboratory standard Institute (CLSI) helped in further identification of bacterial samples.

**Preparation of the standard turbidity (0.5 Mc Farland)**

0.5 ml of 1.175% barium chloride solution with 2 molecules was poured into a 100 ml volumetric flask. 1% sulfuric acid was then added until its volume reached 100 ml. This solution was stable for 6 months at room temperature in the dark. It was stored in a sealed tube whose diameter was the same as the diameter of the pipe used to maintain the microbial suspension. The medium used in antibiogram was Mueller-Hinton. The PH of this medium was between 7.2 and 7.4 and its thickness was about 4 mm.

**Preparation of Bacterial Suspension**

A sample from microbial colonies whose heights were about 3 to 4 mm was taken using a loop. It was poured into the tube containing normal saline and mixed to form a liquid suspension to obtain the standard size of turbidity (half Mcfarland). If the suspension was less or much concentrated than the normal concentration, the diameter of inhibition zone would be larger and smaller, respectively.

**Methods of Conducting the Test**

Dip a sterile swab in the bacterial suspension. Extract the residual material from the solution by pressing the swab to the side of the tube. Then, rub the surface of the plate 3 times. Rotate the swab around the internal side of the plate. Then, put the swab into disinfectant. If the culture plates were kept at room temperature too long, bacteria replicate before laying disks. As a result, the inhibition zone would be smaller. Fifteen minutes after growth of the culture, put the disks on the surface of the medium with a sterile forceps and secure the disks in place using the tip of the forceps.

Put the plates at 35 °C for about 16 to 18 hours. Then, measure the diameter of the inhibition zone in millimeters using an exact ruler. Then, assess the sensitivity and resistance of bacteria to antibiotics. Finally, compare the assessment results with the objectives of the study. The data analysis was done using the statistical software SPSS version 18.

## Results

 In this study, a total of 3366 clinical samples were collected from the patients with acute leukemia hospitalized in hematology and oncology ward. The samples included blood (n=976), urine (n=2190), and wound (n=200) cultures. A positive culture result was found in 760 (22.5%) of samples. The frequency of culture positive samples were 185 (18.95), 503 (22.96%) and 72 (36%) in blood, urine, and wound cultures, respectively. The microorganism isolated from the blood, urine and wound cultures of patients with acute leukemia are shown in Table 1. E. coli was the most common bacteria isolated from the blood cultures (63 samples, 34%). Other common bacteria included Stenotrophomonas Maltophilia, Staphylococcus Epidermidis and Staphylococcus Aureus, respectively. E. coli was the most frequently isolated bacteria from the urine cultures (161 samples, 32%). Other common isolated organisms from the urine culture, in descending order, were as follow: Klebsiella, Enterobacter and Proteus.

**Table 1:** Distribution of bacterial isolates from blood, urine and wound culture of patients with acute leukemia

	**E.coli**	**Staph Epidermidis**	**Staphs Aureus**	**Other Staphlococcus**	**Strepto coccus**	**Klebsiella**	**Pseudomonas**	**Proteus**	**Acinetobacter**	**Enterobacter**	**Enterococcus**	**Stenotrophomonasmaltophilia**	**Total**
Positive Blood culture													
ALL^1^	44	17	7	1	-	-	2	-	-	4	-	37	116
AML^2^	19	10	10	1	-	-	3	-	-	6	-	15	69
Total	63	27	17	2	-	-	5	-	-	10	-	52	185
Positive Urine culture													
ALL	102	24	8	-	-	65	4	27	-	49	15	-	294
AML	59	26	3	-	-	50	2	23	-	36	10	-	209
Total	161	50	11	-	-	115	6	50	-	85	25	-	503
Positive wound culture													
ALL	4	12	16	-	**-**	2	1	1	7	-	-	-	43
AML	2	9	9	-	**-**	3	1	2	3	-	-	-	29
Total	6	21	25	-	**-**	5	2	3	10	-	-	-	72

ALL: Acute Lymphocytic Leukemia, AML: Acute Myeloid Leukemia

The most prevalent bacteria isolated from the patients’ wound cultures was Staphylococcus Aureus (25 samples, 35%), following by Staphylococcus Epidermidis, Acinetobacter and E-coli. Organism most commonly isolated from the blood culture of male patients was Stenotrophomonas Maltophilia (30/82, 36%). However, E. coli was the most common cause of bacteraemia in females (45/103, 43%).Organism most commonly isolated from the urine culture of male and female patients was E. Coli (76/240, 31%).

## Discussion

 Advances in early cancer diagnosis and new treatment strategies have improved the prognosis of cancer patients over the last decade. Unfortunately, severe infections continue to be among the important complications contributing significantly to the morbidity and mortality of these patients_._^9^^,^^10^ There are limited data regarding the epidemiology and antibiotic resistance of microorganisms isolating from the clinical samples of patients with acute leukemia in our region. Cancer patients are exposed to different infections due to the weakness of their immunity system and administration of cytotoxic drugs.^11^^,^^12^ Blood stream infections resulting from gram-negative bacilli have occurred throughout the invasive treatment of cancer patients.^13^^-^^15^ Data obtained from extensive studies carried out in big cancer research institutes in the United States and Europe show that Enterobacteriaceae include 65 to 80 percent of registered gram-negative infections. In recent years, a significant increase in the resistance of antibiotics in gram-negative bacilli has been observed.^16^^-^^17^ An increase in the problems related to antibiotic resistance often stems from an incorrect and widespread use of anti-microbial compounds in the hospitals and in the society. Reliable figures necessary for controlling the incidence of resistant pathogens are available in the developing countries.

Moreover, in case of no decline in the incorrect consumption of drugs, the bacterial species resistant to different classes of antibiotics will increase.

In this cross-sectional study, a total of 760 isolates were cultured from 3366 clinical samples of patients with acute leukemia hospitalized in hematology and oncology ward. Of these, a total of 185 (18.95%) blood, 503 (22.96%) urine and 72 (36%) wound samples were culture-positive, respectively.

**Table 2:** Antibiotic sensitivity and resistance rates of common pathogens isolates from blood cultures

**Antibiotic**	**Staphylococcus Epidermidis**		**E.coli**	**Staph. Aureu** 1	**Steno.** 2	**Pseudo.** 3	**Strept.** 4	**Entero.** 5	**other staph**
**Pipracillin/tazobactam**	S	-	19	-	-	4	-	8	-
	R	-	-	-	-	-	-	-	-
**Ampicillin/Sulbactam**	S	-	25	-	-	4	-	9	-
	R	-	9	-	-	-	-	2	-
**Ciprofloxacin**	S	17	36	14	45	-	9	10	1
	R	6	12	8	-	-	1	3	1
**Erythromycin**	S	11	-	12	-	-	6	-	1
	R	4	-	5	-	-	2	-	1
**Clindamycin**	S	10	-	13	-	-	6	-	1
	R	4	-	5	-	-	2	-	1
**Gentamycin**	S	12	26	12	-	4		-	1
	R	5	-	5	-	-	2	-	1
**Vancomycin**	S	18	-	16	-	-	8	-	2
	R	-	-	-	-	-	-	-	-
**Ceftazidim**	S	-	10	-	-	5	-	-	-
	R	-	8	-	-	3	-	2	-
**Amikacin**	S	-	-	-	30	-	-	-	-
	R	-	10	-	-	-	-	2	-
**Ceftriaxon**	S	-	18	-	37	4	-	-	-
	R	-	13	-	12	4	-	3	-
**Rifampicin**	S	15	-	9	-	-	6	-	1
	R	-	-	-	-	-	-	-	-
**Chloramphenicol**	S	9	-	12	-	-	6	-	1
	R	-	-	-	-	-	-	-	-
**Cotrimoxazole**	S	-	24	-	28	3	5	-	-
	R	11	-	11	21	3	5	4	1
**Imipenem**	S	-	50	-	-	4	-	10	-
	R	-	-	-	-	-	-	-	-

1 **:** Staphylococcus Aureus,

2 **:** Stenotrophomonas Maltophilia,

3 **:** Pseudomonas,

4 **:** Streptococcus,

5 **:** Enterobacter

Various results were obtained from the studies conducted on the prevalence of bacteremia in patients with cancer.^8^^-^^11^ Based on the results obtained from other studies, the rates of infections in the samples of cancer patients vary from 5.7% to 44%.^15^^-^^17^ One study reported that 18% of patients with febrile neutropenia were positive blood-culture.^9^ Ecoli was the most common microorganism isolated from the samples of these patients. In this study, the most frequent pathogen isolated from the blood culture of leukemia patients was E-coli (34%). In many studies conducted on patients with immune deficiencies, the E-coli bacteria were reported as the most common cause of bacterial infections^18^^-^^22^ One study reported that Ecoli was the most common microorganism isolated from the samples of the patients with neutropenic fever.^9^ Another study found that coagulase-negative staphylococci (32%), Staphylococcus Aureus (12%), E-coli (7%) and Enterococcus (6%) were the causes of infection among samples of the neutropenic patients.^8^ In a study conducted by Prabhgsh et al. (2010) in India, it was reported that the most common causes of infection in Leukemia patients were Pseudomonas SPP bacterium (30.37%) and Staphylococcus Aureus (12.6%).^22^

In a study conducted by Chen CY et al. (2009) in Taiwan, 853 patients (65%) had neutropenia. The Gram-negative bacteria were most common among patients with neutropenia (60%) including E-coli (12%), Klebsiella Pneumonia (10%), Acinetobacter Calcoaceticus Baumannii (6%) and Stenotrophomonas Maltophilia (6%).

**Table 3:** Antibiotic sensitivity and resistance rates of common pathogens isolates from urine cultures

**Antibiotic**	**Staph.** 1 ** Epidermidis**		**E.coli**	**Staph** 2 ** Aureus**	**Klebsiella**	**Pseudo.** 3		**Proteus**		**Entero** ***.*** 4		**Enterococcus**	
**Pipracillin/tazobactam**	S	-	57	-	42		2		16		31		-
	R	-	-	-	-	-		-		-		-	
**Ampicillin/Sulbactam**	S	-	45	-	35	2		18		42		-	
	R	-	32	-	8	-		5		6		-	
**Ciprofloxacin**	S	31	110	8	61			29		51		10	
	R	10	20	4	7	-		4		8		7	
**Clindamycin**	S	14	-	5	-	-		-		-		-	
	R	9	-	2	-	-		-		-		4	
**Gentamycin**	S	14	42	6	43	3		12		34		-	
	R	30	-	7	-	-		-		-		5	
**Vancomycin**	S	32	-	11	-	-		-		-		18	
	R	-	-	-	-	-		-		-		-	
**Ceftazidim**	S	-	52	-	48	4		20		44		-	
	R	-	25	-	8	2		6		6		-	
**Amikacin**	S	-		-	-	-		-		-		-	
	R	-	29	-	8	-		5		8		-	
**Ceftriaxon**	S	-	49	-	35	3		22		38		-	
	R	-	27	-	9	3		7		11		-	
**Cotrimoxazole**	S	-	42	-	30	3		19		32		4	
	R	45	-	10	-	-		-		-		20	
**Imipenem**	S		122	-	87	6		42		62		-	
	R	-	-	-	-	-		-		-		-	
**Nitrofurantion**	S	18	104	5	76	-		34		53		-	
	R	-	15	-	12	-		11		8		-	
**Nalidixic acid**	S	-	74	-	70	-		30		47		-	
	R	-	21	-	14	-		17		10		-	

1 **:** Staphylococcus Epidermidis,

2 **:** Staphylococcus Aureus,

3 **:** Pseudomonas,

4 **:** Enterobacter

Among the Gram-positive bacteria, coagulase-negative Staphylococci (19%) and Staphylococcus Aureus (4%) were the most common gram-positive pathogens.^23^ The difference between the results obtained from the studies conducted on this issue may be due to differences in the pattern of antibiotic medications, different health and hygiene issues and differences in underlying diseases in those countries.

In this study, the highest infection rates in blood cultures were caused by Stenotrophomonas Maltophilia (36%) and E-coli (43%) in males and females, respectively. The highest rates of infection in positive urine cultures of male and female patients were caused by E-coli (31%, 32%, respectively). The highest rates of infection in positive wound cultures of male and female patients were caused by Staphylococcus Epidermis (37%) and Staphylococcus Aureus (42%), respectively. In the present study, 116 (63%) out of 185 patients with lymphoblastic leukemia (ALL) had positive blood cultures, 294 (58%) out of 503 had positive urine cultures and 43 (59%) out of 72 had positive cultures. The highest rates of infection were observed in the samples of the patients with acute leukemia.

This result is consistent with those obtained from the studies conducted in our country and other parts of the world. In a study conducted by Cortes JA et al. (2009) in Columbia, ALL cases had the highest rates of infection among hematologic malignancies (54 of 137 ALL cases).^11^

**Table 4:** Antibiotic sensitivity and resistance rates of common pathogens isolates from wound cultures

**Antibiotic**	**Staph.** 1 ** Epidermidis**		**E.coli**	**Staph.** 2 **us Aureus**	**Klebsiella**		**Pseudo.** 3		**Proteus**		**Acinetobacter**	
							
**Pipracillin/tazobactam**	S	-	2	-		2		1		1		2	
	R	-	-	-		-		-		-		-	
**Ampicillin/Sulbactam**	S	-	1	-	2		-		1		2	
	R	-	1	-	-		-		-		2	
**Ciprofloxacin**	S	7	2	8	2				1		2	
	R	5	2	6	2		1		1		3	
**Clindamycin**	S	7	-	5	-		-		-		-	
	R	2	-	4	-		-		-		-	
**Gentamycin**	S	5	2	6	2				1		2	
	R	3	-	4	-		-		-		-	
**Vancomycin**	S	9	-	17	-		-		-		-	
	R	-	-	-	-		-		-		-	
**Ceftazidim**	S	-	2	-	2		1		1		3	
	R	-	-	-	-		-		-		2	
**Amikacin**	S	-	-	-	-		-		-		-	
	R	-	1	-	1		1		1		2	
**Ceftriaxon**	S	-	1	-	2		1		1		2	
	R	-	2	-	2		1		1		3	
**Rifampicin**	S	10	-	9	-		-		-		-	
	R	-	-	-	-		-		-		-	
**Cotrimoxazole**	S	-	2	-	2		-		1		3	
	R	11	2	16	2		1		1		-	
**Imipenem**	S	-	6	-	5		2		3		9	
	R	-	-	-	-		-		-		-	

1 **:** Staphylococcus Epidermidis,

2 **:** Staphylococcus Aureus,

3 **:** Pseudomonas

In the present study, all microorganisms with positive blood cultures were susceptible to Ciprofloxacin. In a study conducted by M. Mizanur Rahman et al. in Bangladesh, it was concluded that prophylaxis with Fluoroquinolones (such as ciprofloxacin) is effective against bacterial infections and reduces bacterial infection. As a result, it delays onset of fever in induced neutropenic fever caused by chemotherapy .^19^ In the present study, resistance to Vancomycin was not observed in blood cultures of the patients; however, 44 sensitive cases of bacteria to this broad-spectrum antibiotic were observed. In one study, the isolated Streptococci showed moderate sensitivity to common beta-lactam drugs such as Penicillin B, Ampicillin groups and their alternative treatments, namely.

Azithromycin.^24^ In addition to Vancomycin, resistance to Fluoroquinolones, Imipenem and Meropenem were not observed among Staphylococci and other gram-positive bacteria.^24^^,^^25^ More than 50% of the isolated E.coli, Pseudomonas and Klebsiella showed multiple resistances to broad-spectrum Cefepime, Ceftriaxone, Imipenem and Meropenem antibiotics.^6^

These results are similar to those obtained in our study. In the present study, the highest level of antibiotic resistance was to Cotrimoxazole in all microorganisms with positive blood cultures and positive urine cultures. Meanwhile, no resistance to Vancomycin and Imipenem was reported. Like the study conducted by Irfan et al. (2008) in Pakistan, no resistance to Imipenem or Meropenem was observed.^9^

The highest rate of sensitivity and the highest level of resistance in the microorganisms with positive wound cultures were to Imipenem and Cotrimoxazole, respectively. No resistance to Vancomycin was reported. In another study, similar results were obtained in which the resistance of Gram-positive bacteria to the Vancomycin antibiotic was low. Moreover, resistance of Gram-positive and Gram-negative bacteria to Ciprofloxacin, Imipenem, Amikacin and Ceftazidim was low.^26^

According to the results obtained from this study and lack of adequate number of studies on assessing specific infections in the patients with leukemia, it is necessary to conduct appropriate studies on this issue in specific conditions in our country. These studies should aim to identify the common infections and rate of antibiotic sensitivity in the patients with leukemia. The results obtained from this research should be used for preventive and therapeutic measures in patients with leukemia. The high level of resistance observed in this study highlights the need for reviewing the model of resistance in anti-microbial agents administered to patients under treatment. The high level of resistance observed in this study highlights the need for reviewing the model of resistance in anti-microbial agents administered to under-treatment patients to manage their treatment more effectively. Precise monitoring of conditions in which the prescribed anti-microbial agents strengthen resistance is deemed important.

## CONCLUSION

 In this study, of 3,366 samples taken from patients with acute leukemia, 760 (22.5%) were culture positive. E-coli was the most common cause of infection in blood and urine cultures while Staph Aureus was the most common cause of infection in wound cultures. The findings of this study can be used in clinics for more accurate diagnosis and more effective treatment, as well as prevention of infection in cancer patients.
